# Ti–Zr–Si–Nb Nanocrystalline Alloys and Metallic Glasses: Assessment on the Structure, Thermal Stability, Corrosion and Mechanical Properties

**DOI:** 10.3390/ma12091551

**Published:** 2019-05-12

**Authors:** Camelia Gabor, Daniel Cristea, Ioana-Laura Velicu, Tibor Bedo, Andrea Gatto, Elena Bassoli, Bela Varga, Mihai Alin Pop, Victor Geanta, Radu Stefanoiu, Mirela Maria Codescu, Eugen Manta, Delia Patroi, Monica Florescu, Sorin Ion Munteanu, Ioana Ghiuta, Nicoleta Lupu, Daniel Munteanu

**Affiliations:** 1 Materials Science Department, Transilvania University of Brasov, Eroilor 29, 500036 Brasov, Romania; camelia.gabor@unitbv.ro (C.G.); daniel.cristea@unitbv.ro (D.C); bedo.tibor@unitbv.ro (T.B); varga.b@unitbv.ro (B.V.), muntean.s@unitbv.ro (S.I.M); ioana.ghiuta@unitbv.ro (I.G.); danielmunteanu@unitbv.ro (D.M.); 2 Faculty of Physics, Alexandru Ioan Cuza University, 700506 Iasi, Romania; velicu.laura@yahoo.com; 3 Department of Engineering Enzo Ferrari, Modena and Reggio Emilia University, 10-41125 Modena, Italy; agatto@unimore.it (A.G.); elena.bassoli@unimore.it (E.B.); 4 Politehnica University of Bucharest, 313 Splaiul Independentei, 060042 Bucharest, Romania; victor.geanta@upb.ro (V.G.); radu.stefanoiu@upb.ro (R.S.); 5 R&D National Institute for Electrical Engineering ICPE-CA Bucharest, 313 Splaiul Unirii, 030138 Bucharest, Romania; mirela.codescu@icpe-ca.ro (M.C.); eugen.manta@icpe-ca.ro (E.M.); delia.patroi@icpe-ca.ro (D.P.); 6 Faculty of Medicine, Transilvania University of Brasov, Eroilor 29, 500036 Brasov, Romania; florescum@unitbv.ro; 7 National Institute of Research and Development for Technical Physics, 700050 Iasi, Romania; nicole@phys-iasi.ro

**Keywords:** biocompatibility, melt spinning, amorphous titanium alloy, thermal stability

## Abstract

The development of novel Ti-based amorphous or β-phase nanostructured metallic materials could have significant benefits for implant applications, due to improved corrosion and mechanical characteristics (lower Young’s modulus, better wear performance, improved fracture toughness) in comparison to the standardized *α*+*β* titanium alloys. Moreover, the devitrification phenomenon, occurring during heating, could contribute to lower input power during additive manufacturing technologies. Ti-based alloy ribbons were obtained by melt-spinning, considering the ultra-fast cooling rates this method can provide. The titanium alloys contain in various proportions Zr, Nb, and Si (Ti_60_Zr_10_Si_15_Nb_15_, Ti_64_Zr_10_Si_15_Nb_11_, Ti_56_Zr_10_Si_15_Nb_19_) in various proportions. These elements were chosen due to their reported biological safety, as in the case of Zr and Nb, and the metallic glass-forming ability and biocompatibility of Si. The morphology and chemical composition were analyzed by scanning electron microscopy and energy-dispersive X-ray spectroscopy, while the structural features (crystallinity, phase attribution after devitrification (after heat treatment)) were assessed by X-ray diffraction. Some of the mechanical properties (hardness, Young’s modulus) were assessed by instrumented indentation. The thermal stability and crystallization temperatures were measured by differential thermal analysis. High-intensity exothermal peaks were observed during heating of melt-spun ribbons. The corrosion behavior was assessed by electrocorrosion tests. The results show the potential of these alloys to be used as materials for biomedical applications.

## 1. Introduction

The evolution of medicine has led to in-depth research regarding the biomaterials used for implants. The aim of the implant design and material is to replace and/or functionalize the lost or diseased biological structure [1]. The bio-functionality and biocompatibility characteristics have been found to play a key role in the performance achieved by any material used for implants in the human body [2].

Nowadays, the metallic biomaterials most often used for surgical implants include 316L stainless steel, cobalt-chromium alloys and titanium and its alloys. It has been shown that both 316L and Cr–Co alloys possess a much higher elastic modulus than bone, leading to insufficient stress transfer to the surrounding bone, thus causing bone resorption and loosening of the implant after some years of implantation (stress shielding) [1,3]. Titanium and its alloys are known to be some of the most researched and developed materials used for implants, due to their adequate properties for these types of applications (e.g., relatively low Young’s modulus, good wear resistance, high strength-to-weight ratio, high corrosion resistance, good osseointegration) [1,4,5,6]. Although the Ti–6Al–4V (α+β alloy) is one of the most used titanium alloys in orthopedic implants, a major concern represents the presence of vanadium in its composition, which is reported to be toxic in its elemental or oxidic form. It was reported that the presence of vanadium ions leads to severe adverse local tissue reactions of the tissue, the transport and accumulation in various organs (liver, lung, spleen, lymph nodes), and cytotoxic effects [7,8,9]. To date, high concentrations of titanium, vanadium and aluminum black debris were discovered in surrounding tissues of the implants [5]. The replacement of vanadium was attempted using Nb in the (α+β) Ti alloy, thus obtaining the Ti–Al–Nb alloy [10] or, even the Ti–Nb–Ta alloys with reported better compatibility compared with Ti–6Al–4V alloy and excellent corrosion resistance [11]. Consequently, the development of proper β-phase titanium alloys, with enhanced mechanical properties in relation to the surrounding bone tissue is desired [4,12,13]. These are obtained by the addition of β-stabilizers which simultaneously should exhibit good biocompatibility, apart from the effect on the crystalline structure.

Furthermore, β–Ti alloys and Ti-based bulk metallic glasses can exhibit a unique combination between high yield strength and lower Young’s modulus [14]. Relatively similar mechanical properties to human bone were found to be possessed by non-toxic porous bulk glassy Ti_42_Zr_40_Ta_3_Si_15_ alloy, produced by a combination of rapid solidification and powder metallurgy techniques. The obtained amorphous alloy has presented Young’s modulus of about E = 52 GPa (relatively close to that of human bone, E = 10–30 GPa), while pure titanium and the Ti–6Al–4V alloy usually have an elastic modulus around E = 110–120 GPa [14,15].

Ti–Nb–Hf compositions of β-type titanium alloys were developed by M. Gonzalez et al., using β-stabilizing alloying elements, such as Nb, Ta, Zr, and Hf, with the objective of obtaining a lower elastic modulus (70 to 90 GPa reported). Lower values of Young’s modulus could be obtained after cold working processes [16]. A closer value (44 GPa) to that of the cortical bone was obtained for 99% cold working condition of the Ti–16.2Hf–24.8Nb–1Zr alloy. Moreover, one of the alloys exhibited shape memory effect properties, more precisely a reversible phase transformation due to the presence of thermoelastic martensitic α’’ plates inside the β grains [17].

Various types of metallic glasses (Zr-based [18,19,20], Mg-based, Zn-based, Ca-based, and Ti-based) alloying systems were studied due to their higher strength, lower Young’s modulus, better wear and corrosion resistance, compared to their standardized counterparts [21]. Although regarding the Ti-based metallic glasses reports on systems such as Ti–Zr–Si [15], Ti–Zr–Cu–Pd [20], Ti–Zr–Ni–Be [19] and others can be found in the specific literature, there is a limited number of articles referring to the Ti–Zr–Nb–Si metallic glasses obtained by melt-spinning [15,22,23]. The possibility to obtain Ti–Zr–Nb–Si thin films metallic glasses by the sputtering technique was also reported [24].

Corrosion and passivation behavior of metallic glasses free of toxic elements, namely Ti_75_Zr_10_Si_15_ and Ti_60_Zr_10_Nb_15_Si_15_, produced as melt-spun ribbons by Abdi et al. for biomedical applications was also studied [21]. Mechanical properties investigations of Ti_75_Zr_10_Si_15_ revealed that Nb has the main effect of decreasing the Young’s modulus of the crystalline alloy as well as its hardness, which is due to the stabilization of a significant fraction of a β-type phase [23].

The potential of similar types of alloys is presented in this paper, namely regarding Ti-based amorphous metallic structures which contain non-harmful elements, such as Zr, Nb, and Si, in various proportions. These alloys were obtained by melt-spinning, considering the ultra-fast cooling rates this method can provide. However, one must consider the fact that, to date, metallic glasses can be obtained in relatively small quantities, and with significantly reduced dimensions (5–6 mm diameter rods, in best case scenarios), which makes their use as orthopedic components significantly difficult.

Hence, our attention was aimed at the possibility to develop Ti-based metallic glasses, with emphasis on the final material properties, after recrystallization. The devitrification phenomenon which occurs during heating could contribute to lower input power during additive manufacturing technologies, such as powder bed fusion. The effect of the chemical composition variation and cooling rate during melt-spinning, related to the structural development, thermal stability, mechanical properties, and electrocorrosion behavior was of interest.

## 2. Materials and Methods

### 2.1. Materials Design

The alloy ingots with nominal composition Ti–Zr–Si–Nb were prepared by vacuum arc melting (MRF ABJ 900; MRF—Materials Research Furnaces, Allenstown, NH, USA) in a water-cooled copper crucible under an argon atmosphere. Bars and lumps of the pure metals (Ti = 99.9 wt%, Si = 99.9 wt%, Nb = 99.8 wt%, Zr = 99.8 wt%) were weighed to obtain the compositions presented in Table 1 for a 100-grams charge.

To ensure the stability of the electric arc between the W-Th electrode and the metallic charge, a 5×10^−3^ mbar vacuum level was obtained in the chamber, followed by purging with argon for 20 min. In order to achieve a homogeneous distribution of the elements each alloy ingot was re-melted five times. The actual density of the alloy ingots was determined by hydrostatic weighing. The density is smaller than the theoretical calculated additive value with an assimilation degree for the three alloys, Ti_60_Zr_10_Si_15_Nb_15_, Ti_64_Zr_10_Si_15_Nb_11_, and Ti_56_Zr_10_Si_15_Nb_19_ of 99.95%, 99.8% and 99.65% respectively. This difference could be explained by vaporization loss and potentially the formation of small pores inside the ingots. The three alloys differ in the Ti versus the Nb content, one substituting the other in certain amounts. The Zr and Si content was kept the same for all the compositions. The proportion of Si was chosen in relation to the Ti–Si phase diagram, where, for close to 15% at. Si, the β–Ti+Ti_5_Si_3_ eutectic is formed, at 1340 °C, hence this composition should give adequate results in terms of glass formation. The varying elements (Ti and Nb) have the following characteristics, in elemental form: Young’s modulus Ti = 102.7 GPa vs. Nb = 105.0 GPa, melting temperature Ti = 1668 °C vs. Nb = 2477 °C, density Ti = 4.50 g/cm^3^ vs. Nb = 8.57 g/cm^3^. However, the most important aspect is the β-isomorphous characteristic of Nb in relation to Ti (due to their very close atomic radii), which translates in increased solubility of Nb in Ti. The addition of Nb to the Ti–Zr–Si alloy was reported to lower the elastic modulus of the alloy [23]. Consequently, the proportion between Ti and Nb was changed, in order to study its effect on the mechanical characteristics, along with the structural development, thermal stability, and biocorrosion response.

The titanium-based thin ribbons were obtained by melt-spinning, a well-known fast-cooling technique that usually produces ribbons or foils with amorphous or nanocrystalline structure [25,26,27]. For optimal results, the heat transfer coefficient at the melt-cooling interface has to be considered. In the case of melt spinning, the cooling medium is the material of the rotating disk—a rotating copper wheel with a high thermal conduction coefficient.

Two different melt-spinning installations were used, in order to observe the influence of the cooling rate on the final alloy structure and properties. The relevant differences between the two melt-spinning processes (MS1 and MS2) were:

1. MS1—ZrO_2_ crucibles, crucible outlet diameter 1 mm, positioned at 1 mm distance from the copper disk. The copper disk rotation speed was 2600 rpm (resulting in a peripheral speed of 28 m/s). The entire setup (induction coil, crucible, copper disk) was introduced in a steel rectangular enclosure, which was purged with argon (P = 1.5 bar), to avoid oxidation.
2. MS2—BN crucibles, crucible outlet diameter 0.8 mm, positioned at 0.5 mm distance from the copper disk. The copper wheel had a peripheral speed of 36 m/s. The chamber was evacuated to 10^−6^ mbar, followed by argon purging.

### 2.2. Characterization of Ti-based Metallic Structures

The morphology and chemical composition of the samples were obtained using a scanning electron microscope (SEM, Quanta, Thermo Fisher Scientific–FEI, Hillsboro, OR, USA) operated in high vacuum mode, equipped with an X-ray energy dispersion spectroscopy system (X-EDS, INCA Oxford Instruments, Abingdon, UK) for chemical micro-analysis.

The structural analysis of the Ti–Zr–Si–Nb alloy ribbons was made using the BRUKER AXS D8 DISCOVER diffractometer (Bruker, Karlsruhe, Germany) configured in θ–2θ geometry, in parallel radiation provided by the primary optic beam formed by the X-ray tube with Cu anode, followed by a 0.6 mm Göbel mirror. The samples were placed on a commercially available quartz sample holder (cut so as not to generate reflections) and the diffractogram was recorded using the LynxEye 1D multichannel detector.

The crystallization temperatures of the samples were studied on an F3 Jupiter STA/TG/DTA (NETZSCH, Selb, Germany), with a heating/cooling rate of 10 K/min in argon atmosphere, up to the maximum temperature of 1803 K, and data processing was done using the NETZSCH Proteus Analysis software (v. 5.2.1/2011, Netzsch, Selb, Germany). The calibration of the instrument was done before each measuring session.

The electrochemical measurements were performed for all bulk alloy samples in a conventional electrochemical cell containing three electrodes: discs of alloys as a working electrode, a platinum wire as an auxiliary electrode, and Ag/AgCl/3.5 M KCl as a reference electrode. The working electrode was prepared by connecting an electric lead to one circular surface of the alloy disc and then embedding all parts except the second circular surface in an epoxy-acrylic resin. After this, the specimens were ultrasonically cleaned in distilled water and rinsed with ethanol, and then used for electrochemical evaluation.

The potentiodynamic methods and electrochemical impedance spectroscopy (EIS) were carried out both in 0.9% NaCl solution at room temperature (22 °C) by using a PC-controlled potentiostat PalmSens3 (PalmSens, Houten, The Netherlands) with PSTrace 5.5 software. The corrosion extension of PSTrace software provided the possibility to perform specific types of corrosion measurements and data analysis. The measurements were done in triplicate in each case and average values of all corrosion parameters were calculated along with the computed standard deviation values. Potentiodynamic tests were registered with a 0.002 V/s scan rate and working potential sweep from −1 V to +2 V vs. Ag/AgCl. For electrochemical impedance spectroscopy (EIS) measurements a rms perturbation of 10 mV was applied over the frequency range of 50 kHz–0.1 Hz, with 10 frequency values per frequency decade. Based upon the principles of electrochemical spectroscopy and using the PSTrace 5.5 software with FRA module, the electric equivalent circuit best fitting the experimental data with electrical parameters was inferred.

Some of the mechanical characteristics of the bulk alloys and melt-spun ribbons were assessed by instrumented indentation measurements, which were performed using an NHT^2^ indenter from CSM Instruments/Anton Paar (Pesseux, Switzerland). Hardness and Young’s modulus values were determined from the loading and unloading curves data based on the Oliver and Pharr model [28]. At least 20 indentations, with a Berkovich diamond tip, were performed on the melt-spun ribbons and a minimum of 40 indentations on the bulk material, spaced by 5 µm between imprints. The larger number of indentations on the bulk material is related to the inhomogeneity of the samples in terms of structure, hence of the properties. The maximum applied load was 20 mN, with a loading rate of 40 mN/min and a 100 mN/min unloading rate. The approach speed was 2000 nm/min and the acquisition rate 10Hz.

## 3. Results and Discussions

### 3.1. Structure, Morphology and Chemical Composition

**Figure 1:**
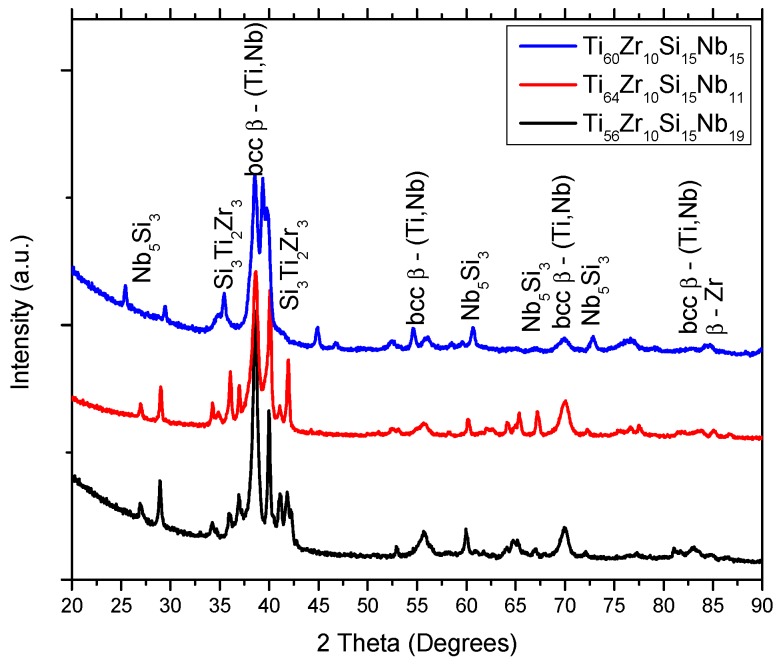
X-Ray Diffraction patterns of Ti–Zr–Si–Nb bulk alloys.

**Figure 2:**
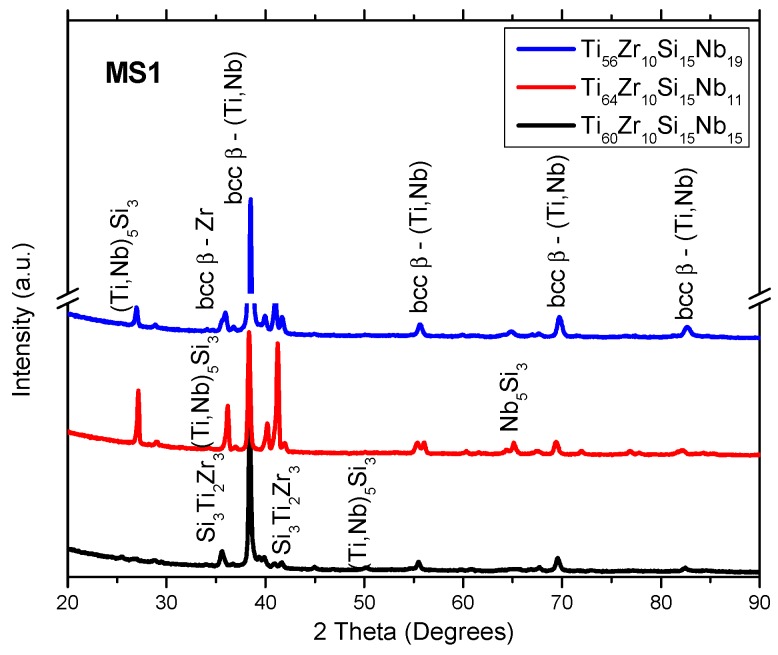
X-ray diffraction patterns of Ti–Zr–Si–Nb alloys obtained with 28 m/s peripheral speed—MS1.

**Figure 3:**
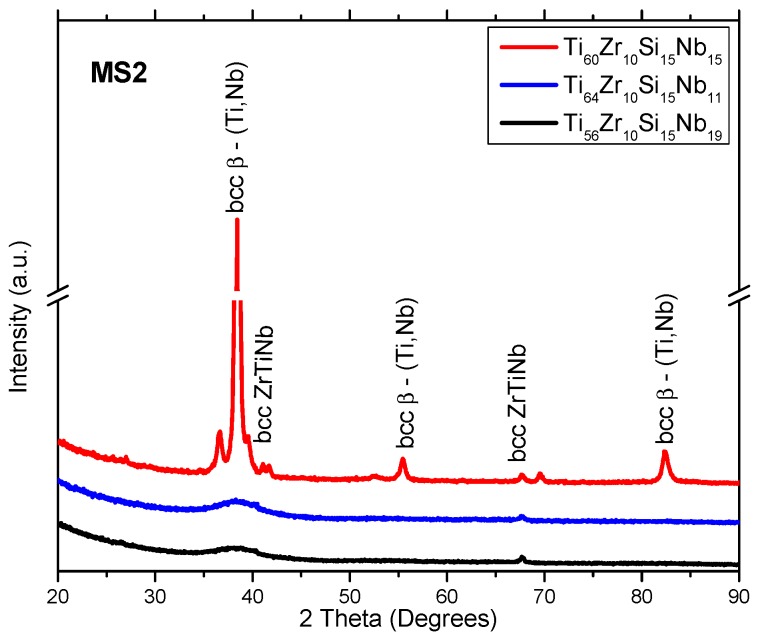
X-ray diffraction patterns of Ti–Zr–Si–Nb alloys obtained with 36 m/s peripheral speed—MS2.

Figure 1, Figure 2 and Figure 3 represent the XRD patterns of the bulk, and melt-spun alloys, separated by composition (Ti_60_Zr_10_Si_15_Nb_15_, Ti_64_Zr_10_Si_15_Nb_11_, Ti_56_Zr_10_Si_15_Nb_19_) and by the melt-spinning conditions, in the case of the ribbons (rotation speed, MS1 (28 m/s) vs. MS2 (36 m/s)). The structure of the bulk alloys consists of the following phases: The predominant bcc β–(Ti,Nb) phase and the silicide compounds: Nb_5_Si_3_ and Si_3_Ti_2_Zr_3_. A great deal of overlap is noticed on the diffraction patterns for the bulk alloys. Hence, the exact attribution of the diffraction peaks would be problematic, also considering the similar angular position of the peaks, exhibited by these compounds. The structure of the melt-spun alloys shows a dependence on the alloy composition. A second dependence on the structural evolution is related to the variable parameters of the melt-spinning processes, i.e., rotation speed/cooling rate. A predominant (high-intensity) peak located at 2θ = 38°, attributed to the bcc β–(Ti,Nb) phase can be observed in both MS1 and MS2 melt-spinning processes. This β phase is noticed at other angular positions, suggesting that this phase is predominant. Consequently, these alloys should be considered β-alloys. Amorphous ribbons were obtained for Ti_56_Zr_10_Si_15_Nb_19_ and Ti_64_Zr_10_Si_15_Nb_11_ alloys (Figure 3) in the MS2 process, due to the higher cooling rate. The broad bands associated with these two compositions, obtained in the MS2 process, are characteristic of amorphous materials. Considering that the germination/crystallization phenomenon does not take place, there are no planes on which the X-ray beam would diffract on. A crystal is composed of periodically arranged atoms in a 3D space. On the other hand, amorphous materials do not possess that periodicity and atoms are randomly distributed in the 3D space. The scattering of X-rays by atoms is the main cause of the appearance of these broad bands.

It is evident that the peripheral speed of the copper wheel significantly affects the cooling speed of the alloy, and consequently, of the final structural arrangement. Even if most of the variants of these alloys could not be obtained in amorphous form, the crystalline structure is evidently fine, as observed in the crystallite sizes (shown in Table 2) estimated with the Scherrer equation for the predominant β phase. One can notice that the crystallite size for the predominant β phase is close to or smaller than 20 nm. This phenomenon of structural refinement has an observable influence on structural stability during heating, as it will be later shown. Other phases found on the diffractograms were predominantly intermetallic silicon compounds: (Ti,Nb)_5_Si_3_ (hexagonal lattice—03-065-3599) and Si_3_Ti_2_Zr_3_ (hexagonal lattice—00-047-1339).

A possible explanation related to the difference in structural evolution for the samples in the MS2 batch, the fact that only two compositions could be obtained in amorphous form, it could be related to the mixing enthalpy (ΔH_mix_ (kJ/mol)) and the relative difference between the diameters of the alloying elements atoms (δ parameter). Concerning the mixing enthalpy, the calculated values are the following: Ti_60_Zr_10_Si_15_Nb_15_ ΔH_mix_ = −28.4 kJ/mol, Ti_64_Zr_10_Si_15_Nb_11_ ΔH_mix_ = −33.3 kJ/mol, Ti_56_Zr_10_Si_15_Nb_19_ ΔH_mix_ = −32.4 kJ/mol. These values are characteristic for alloys which can be amorphized relatively easily. However, if we consider the δ parameter, there are clear differences between the three variants of Ti-based alloys: Ti_60_Zr_10_Si_15_Nb_15_ δ = 8.02, Ti_64_Zr_10_Si_15_Nb_11_ δ = 8.63, Ti_56_Zr_10_Si_15_Nb_19_ δ = 8.61. Considering these calculated values, only the Ti_64_Zr_10_Si_15_Nb_11_ and Ti_56_Zr_10_Si_15_Nb_19_ alloys are in the potentially amorphous materials’ domain, while the Ti_60_Zr_10_Si_15_Nb_15_ is located outside of this region, hence the nanocrystalline structure for the latter composition would be expected, and not the amorphous one.

**Figure 4:**
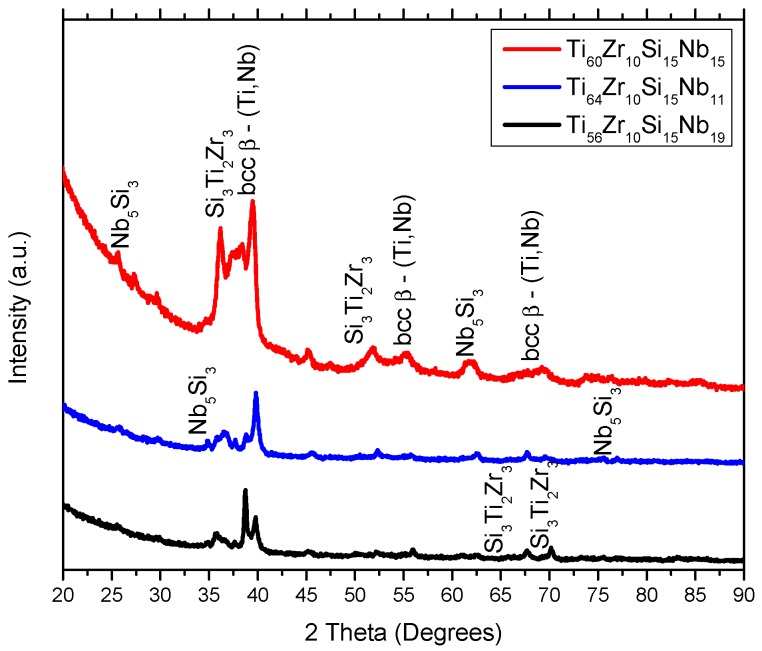
X-ray diffraction patterns of Ti–Zr–Si–Nb alloys obtained with 36 m/s peripheral speed—MS2, after heat treatment.

The melt-spun ribbons from the MS2 batch were subjected to a heat treatment in argon atmosphere, past the phase transformation temperatures noticed on the DTA curves, up to T_max_ = 800 °C (bellow the allotropic phase transformation between α-Ti and β-Ti, which occurs at 890 °C, when no alpha or beta-stabilizer elements are present), presented in Section 3.4. The diffraction patterns for these samples are presented in Figure 4. The Ti_60_Zr_10_Si_15_Nb_15_ and Ti_64_Zr_10_Si_15_Nb_11_ alloy compositions exhibit almost identical structures, compared to the ones exhibited by the melt-spun ribbons (MS1 or MS2): The intermetallic silicon compounds: (Ti,Nb)_5_Si_3_ (hexagonal lattice—03-065-3599) and Si_3_Ti_2_Zr_3_ (hexagonal lattice—00-047-1339), and the bcc β-(Ti,Nb) eutectic. In the case of the Ti_56_Zr_10_Si_15_Nb_19_ composition, a different behavior is observed. At the 34.94, 37.70, 39.84, 52.31, and 75.58° angular positions, the diffraction peaks can be attributed to the hcp α-Ti phase, namely the (100), (002), (101), (102), and (112) directions. This phenomenon could be attributed to the higher Nb content, compared to the other alloy compositions. The presence of beta-stabilizers (in this case Si and Nb) lowers the phase transformation temperature between the bcc β-(Ti /Nb) and the hcp α-Ti phases.

**Figure 5:**
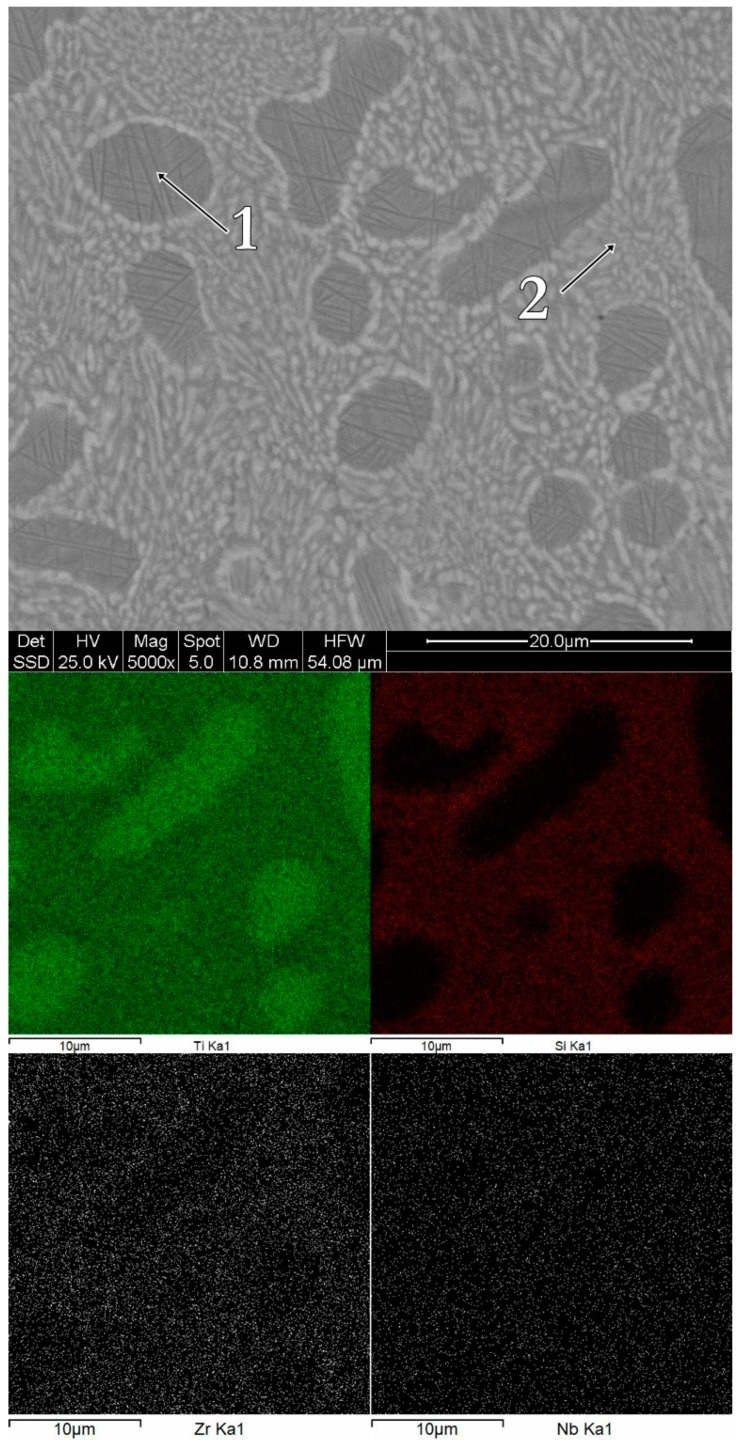
Surface morphology and chemical mapping for the bulk Ti_60_Zr_10_Si_15_Nb_15_ alloy.

**Figure 6:**
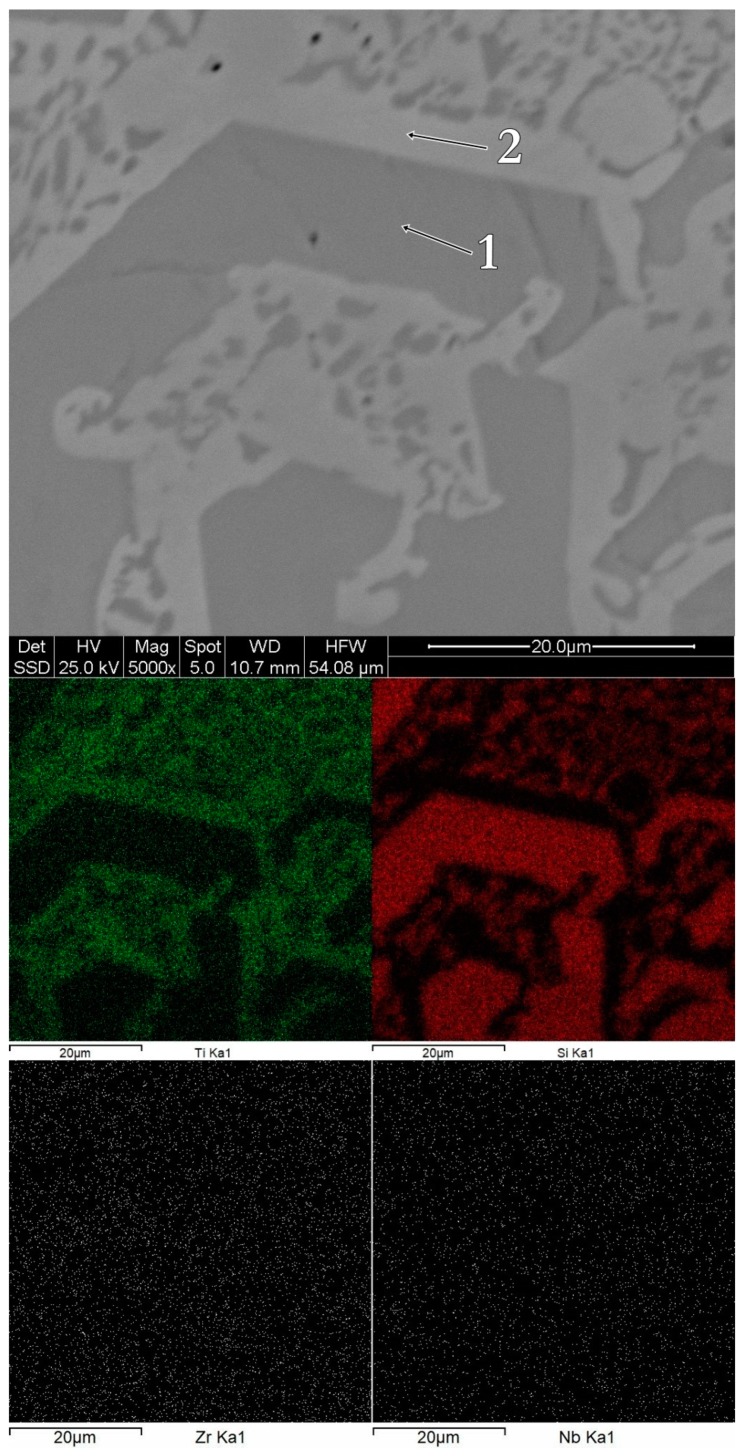
Surface morphology and chemical mapping for the bulk Ti_64_Zr_10_Si_15_Nb_11_ alloy.

**Figure 7:**
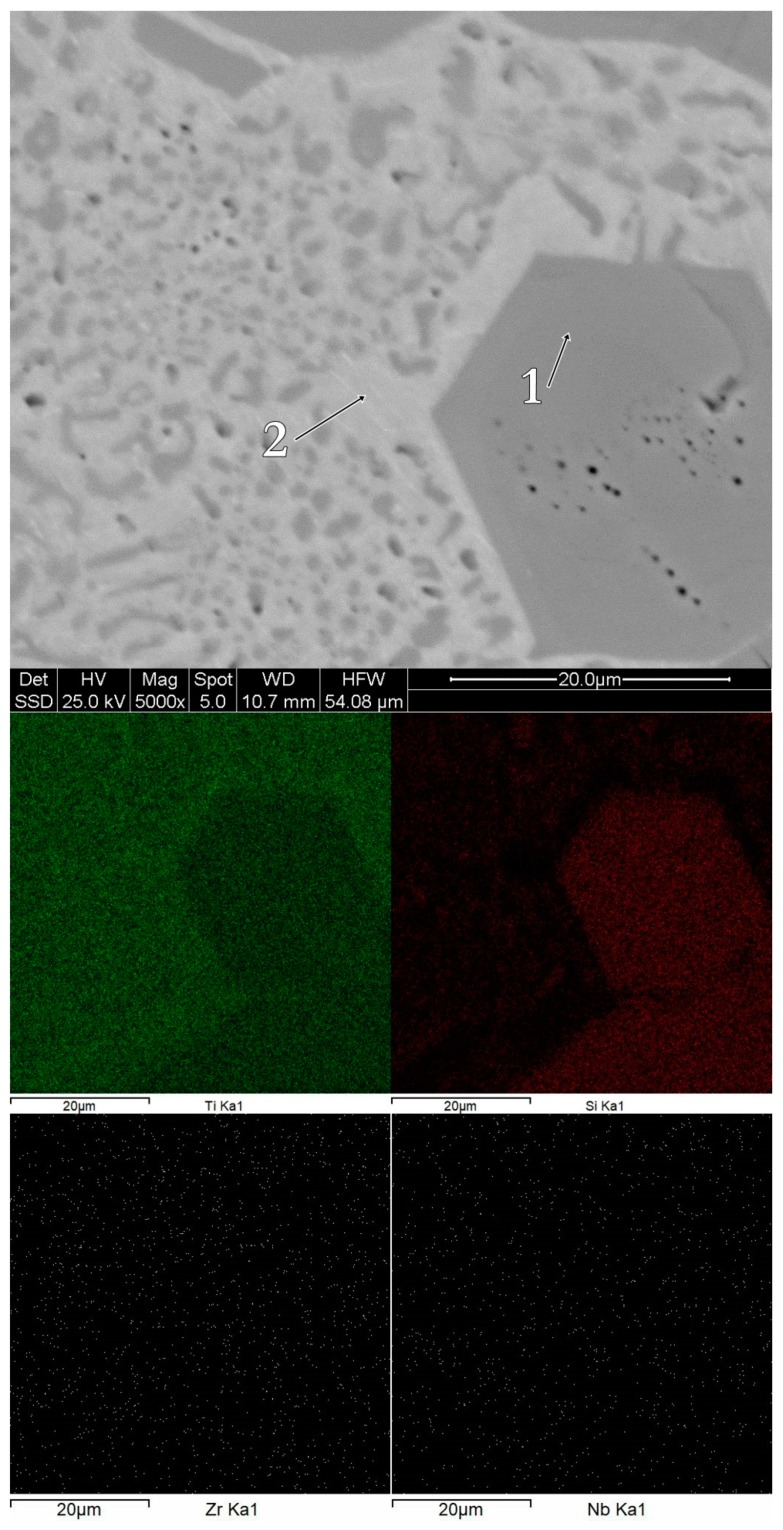
Surface morphology and chemical mapping for the bulk Ti_56_Zr_10_Si_15_Nb_19_ alloy.

The morphology and the chemical composition of the bulk alloys can be observed in Figure 5, Figure 6 and Figure 7. The bulk Ti_60_Zr_10_Si_15_Nb_15_ alloy is characterized by a light matrix-like silicon-rich region, with embedded darker titanium-rich compounds. In the case of this alloy composition, the Ti-rich regions are Zr-depleted, as shown in Figure 5. The bulk Ti_64_Zr_10_Si_15_Nb_11_ alloy exhibits polyhedral crystals, embedded in a lighter matrix. The polyhedral crystals are Si and Zr-rich, depleted in titanium, while the lighter phase represents the β–(Ti,Nb) compound. Similar characteristics are shown by the bulk Ti_56_Zr_10_Si_15_Nb_19_ alloy, with darker silicon-rich regions and lighter titanium-rich regions. The chemical composition for each region is reported in Table 3.

There are clear differences in terms of morphology between the alloy compositions. However, due to the complexity of the quaternary system, it would be hazardous to give definitive conclusions.

The discussion related to the alloy structural development relies on the thermodynamics of the system. Considering the relative novelty of this quaternary composition of Ti-based alloy, the quaternary phase diagram could not be found in the literature. However, if one looks at the binary phase diagrams, some predictions could be made. From the Ti–Zr [29], Ti–Nb [30], and Zr–Nb [31] binary phase diagrams, one can notice that mostly solid solutions are formed between these elements, regardless of the composition and temperature. The presence of solid solutions was evidenced by the XRD analysis, from the bcc–β-Ti,Nb- and the bcc–ZrTiNb-attributed diffraction peaks. The addition of Si to the ternary Ti–Zr–Nb system makes the system significantly complicated. Several silicide compounds can be formed, according to the Ti–Si [32], Zr–Si [33], and Nb–Si [34] binary phase diagrams, namely: Si_2_Ti, SiTi, Si_4_Ti_5_, Si_5_Ti_3_, and SiTi_3_ from the Ti–Si phase diagram; Si_2_Zr, α-SiZr, α-Si_4_Zr_5_, β-SiZr, β-Si_4_Zr_5_, Si_2_Zr_3_, Si_3_Zr_5_, SiZr_2_, and SiZr_3_ from the Zr–Si phase diagram; and Nb_3_Si, α-Nb_5_Si_3_, β-Nb_5_Si_3_, and NbSi_2_ from the Nb–Si phase diagram. The formation of these silicide compounds depends to a great extent on the enthalpy of formation. The diffraction patterns contain peaks for Me_5_Si_3_-type silicide compounds, where Me = Ti, Nb, Zr. The enthalpy of formation at 298K for these silicide compounds is: Ti_5_Si_3_ ΔH^S^ = −573 kJ/mol [35]; Zr_5_Si_3_ ΔH^S^ = −625.4 kJ/mol [35]; Nb_5_Si_3_ ΔH^S^ = −62.2 kJ/mol [36]. Thus, the formation of the Ti_5_Si_3_ and Zr_5_Si_3_ phases should be more favorable. It could be said that the formation of these silicide compounds relies on the competition between the Me elements (Me = Ti, Zr, Nb). Considering the relatively close value for the enthalpy of formation between the Ti_5_Si_3_ and Zr_5_Si_3_ phases, the presence of the Si_3_Ti_2_Zr_3_ compound can be explained, as seen on the X-ray diffraction patterns presented in Figure 1, Figure 2 and Figure 4.

Another aspect that might influence the change in morphology as a function of the chemical composition of the alloys could be related to the particularity of the binary Me–Si phase diagrams, in respect of the very narrow domains associated to the formation of silicide compounds, especially the ones evidenced by the X-ray diffraction structural analysis. The titanium content in the Ti–Zr–Si–Nb alloys, correlated to the titanium content in the Ti–Si binary phase diagram, shows that the three proposed compositions, namely Ti_60_Zr_10_Si_15_Nb_15_, Ti_64_Zr_10_Si_15_Nb_11_, and Ti_56_Zr_10_Si_15_Nb_19_, could be associated to three distinct domains. The Ti_5_Si_3_ silicide compound appears for titanium concentrations between 60.5 and 64.5 at. % [37], from the liquid state, at 2393.9 K [38], while the Ti_5_Si_4_ peritectic appears at a concentration of titanium equal to 55.6 at. % [37], from the liquid+Ti_5_Si_3_ phase, at 2192.6 K [38]. Thus, the Ti_56_Zr_10_Si_15_Nb_19_ composition would be located closer to the peritectic transformation; however, the formation of the Ti_5_Si_4_ would not occur due to the lower Si content. Moreover, the Ti_60_Zr_10_Si_15_Nb_15_ composition would be close to the formation of the Ti_5_Si_3_ silicide, while the Ti_64_Zr_10_Si_15_Nb_11_ composition would be positioned inside but towards the end of the Ti_5_Si_3_ domain.

Consequently, considering the chemical composition presented in Table 3, as well as the hypotheses presented previously, the morphological regions associated to the EDS spectra could be summarized as follows: For the Ti_60_Zr_10_Si_15_Nb_15_ alloy composition, spectrum 1 represents the solid solution composed of Ti+Zr+Nb, while spectrum 2 represents a mechanical mixture of silicide-type phases; for the Ti_64_Zr_10_Si_15_Nb_11_ alloy composition, spectrum 1 represents a mixture of silicide phases, while spectrum 2 represents a Ti+Nb solid solution mixed with silicide phases; and for the Ti_56_Zr_10_Si_15_Nb_19_ alloy composition, spectrum 1 represents silicide phases, while spectrum 2 represents a mixture of solid solution and silicide phases.

**Figure 8:**
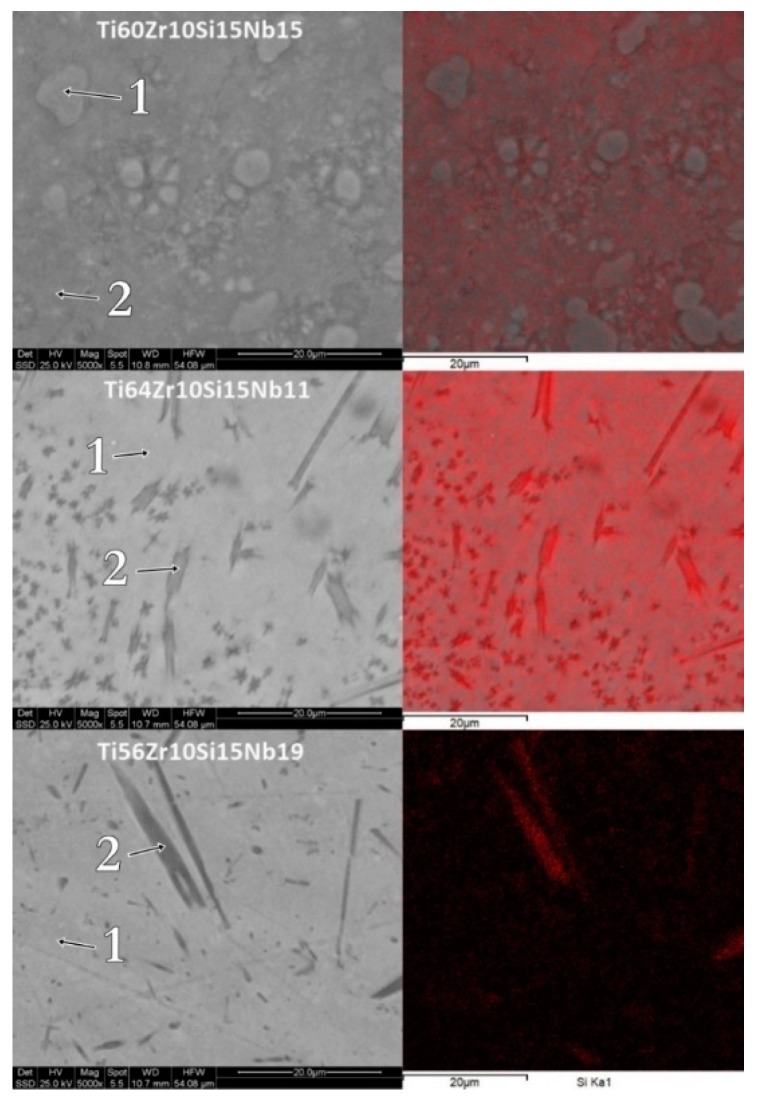
Surface morphology and chemical mapping (Si) for the melt spun alloys, obtained with 28 m/s peripheral speed.

**Figure 9:**
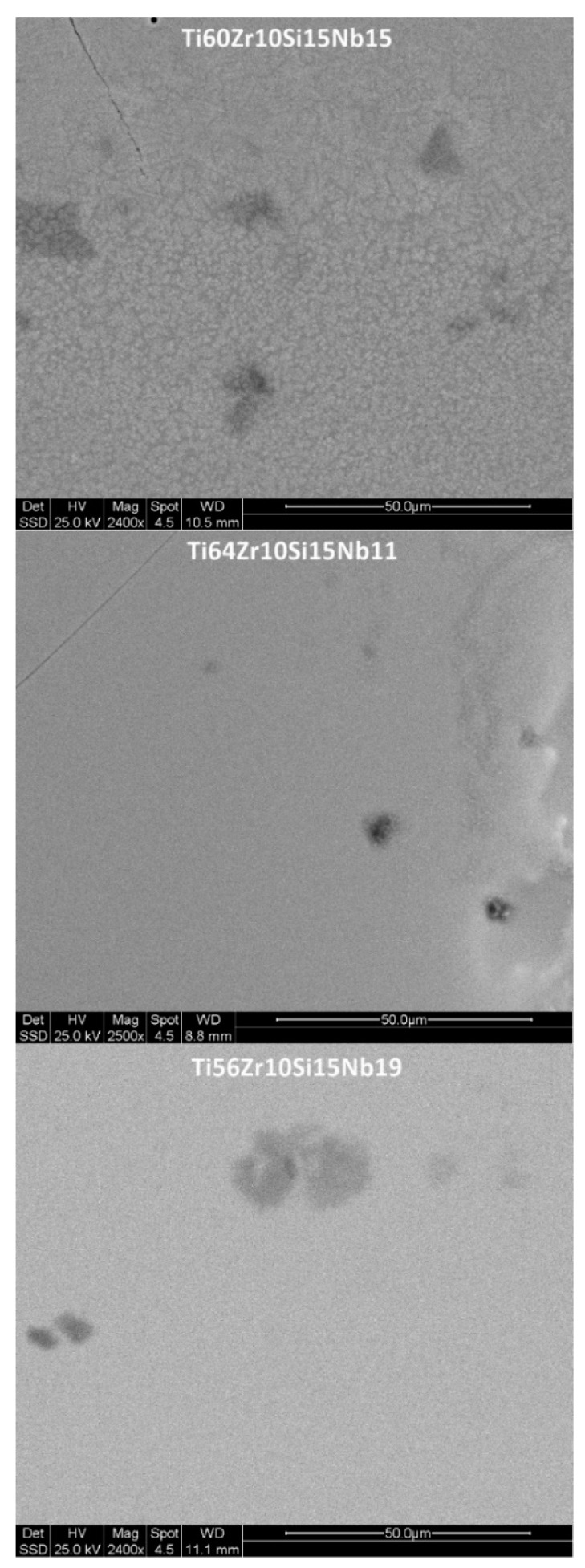
Surface morphology for the melt spun alloys, obtained with 36 m/s peripheral speed.

The morphology and chemical mapping for silicon, for the melt-spun ribbons obtained with 28 m/s peripheral speed, for all three compositions, is shown in Figure 8. The polyhedral crystalline structures, noticed in the case of the Ti_64_Zr_10_Si_15_Nb_11_ and the Ti_56_Zr_10_Si_15_Nb_19_ bulk alloys inside the eutectic matrix, are no longer visible in the case of the melt-spun ribbons, however silicon-rich needle-like grains are visible. This difference in structural development is a direct cause of the increased cooling rate, compared to the cooled copper crucible cast bulk alloys. Due to this increased cooling rate, the nucleation and crystal growth is hindered. Further increasing the cooling rate, as is the case for the MS2 batch of alloys, leads to either nanostructured melt-spun ribbons, where some grains can be observed after mechanical polishing (Figure 9) (Ti_60_Zr_10_Si_15_Nb_15_ alloy), to featureless/amorphous melt spun ribbons, where no discernable chemical composition variation could be observed, which is to be expected, considering that the nucleation is entirely suppressed (Ti_64_Zr_10_Si_15_Nb_11_ and the Ti_56_Zr_10_Si_15_Nb_19_ alloys).

### 3.2. Mechanical Properties

**Figure 10:**
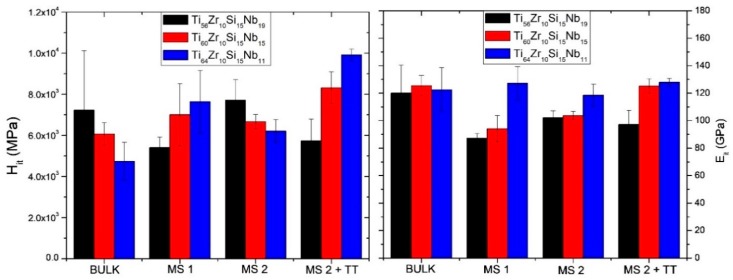
Hardness and Young’s modulus as a function of processing parameters.

Considering that these alloys would be intended for biomedical applications, where the mechanical characteristics are just as important as the biocompatibility and the corrosion resistance, some of the mechanical properties (hardness, Young’s modulus) were assessed by instrumented indentation. The variation of these two characteristics can be observed in Figure 10 and Table 4, as a function of the chemical composition and the processing parameters.

**Figure 11:**
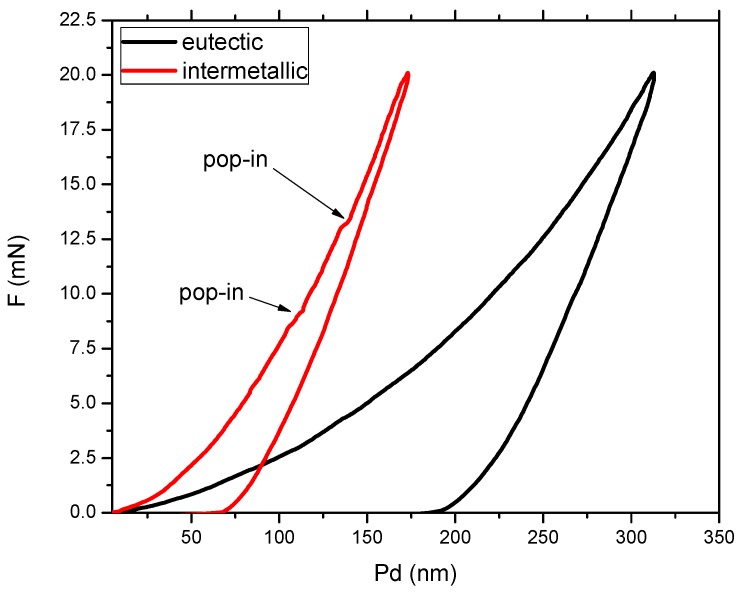
Instrumented indentation loading-unloading curves for the Ti_64_Zr_10_Si_15_Nb_11_ bulk alloy, obtained on the softer eutectic and the harder intermetallic compound.

One particular requirement for alloys which should be used in orthopedic applications is that the material should exhibit a significantly reduced Young’s modulus, as close as possible to the one of the adjacent bone tissue. Depending on the type of bone structure, Young’s modulus can vary significantly: For trabecular bone, between 10–14 GPa, while for cortical bone, between 18–20 GPa [39]. Moreover, the bone modulus varies in magnitude depending on the direction of measurement [1]. The observation of other authors, that with increased Nb content, the elastic modulus is lower, applies to our findings, as well. In terms of hardness, a growth of the hardness values compared to the bulk material can be observed for both MS1 and MS2 ribbons of Ti_64_Zr_10_Si_15_Nb_11_ and Ti_60_Zr_10_Si_15_Nb_15_ alloy, while the alloy with a lower titanium concentration rather shows a decrease of the hardness values (Figure 10). However, one must consider the difference in measuring error, especially for the bulk alloys, which is the direct result of the inhomogeneity of the samples, in terms of structural constituents. The mechanical properties of these constituents were further measured by instrumented indentation on the bulk samples and the results are the following: the silicide constituent exhibits H_it_ 14.09 ± 1.8 GPa, E_it_ = 210.88 ± 13.45 GPa, while the β-Ti,Nb component exhibits H_it_ = 6.04 ± 0.93 GPa, E_it_ = 113.28 ± 12.51 GPa. The measuring error decreases with the refinement of the structure, the MS2 melt-spun ribbons, either as-cast or thermally treated, exhibiting the lowest values. A heat treatment process consisting of heating over the phase transformation temperature followed by in-furnace cooling was also applied to the melt-spun ribbons from the MS2 batch. Only the Ti_64_Zr_10_Si_15_Nb_11_ alloy presented significantly higher hardness compared to bulk material’s hardness value. Further analysis of the Ti_64_Zr_10_Si_15_Nb_11_ bulk alloy sample is presented in Figure 11 where the loading-unloading curves for the structurally different regions are presented. The characteristics of the remaining bulk alloys are similar, hence the loading-unloading curves for the Ti_56_Zr_10_Si_15_Nb_19_ and Ti_60_Zr_10_Si_15_Nb_15_ alloys are not shown, in order to better observe the curve features.

The graph from Figure 11 represents the variation of the applied load as a function of the penetration depth, on two distinct regions, the softer eutectic matrix (black curve) and the harder hexagonal silicide intermetallic compound (red curve). Considering the significant difference in penetration depth for the same applied load, it is obvious that the eutectic matrix (the β-phase) is significantly softer. One can notice certain deflections from the regular path on the loading curve for the intermetallic compound region, which signify “pop-in” events. These steps are an indication that one of several phenomena can occur: Micro-cracks, phase transformations, dislocations nucleation, strain transfer across grain boundaries, all due to the applied load.

If we consider the Hall–Petch (H–P) relation, which helps to conclude that with decreasing crystallite size, the strength/hardness of the questioned material increases, it seems that, concerning the MS1-series samples, the threshold is located above 21 nm crystallite size. This critical threshold concerning the crystallite size, past where the H–P relation is no longer valid, signifies that the samples from batch MS1 can be characteristic of the reverse H–P relation. Below this critical crystallite size, a further refinement of the structure leads to a decrease of the mechanical properties, as can be noticed from the variation of the crystallite size as a function of the hardness (lower crystallite size is related to lower hardness, Table 2 and Table 4). The reverse H–P relation suggests a shift in the dominating deformation mechanisms from dislocation-mediated plasticity to crystallite-boundary-associated plasticity, such as crystallite–boundary sliding, crystallite–boundary diffusion, and crystallite rotation.

**Figure 12:**
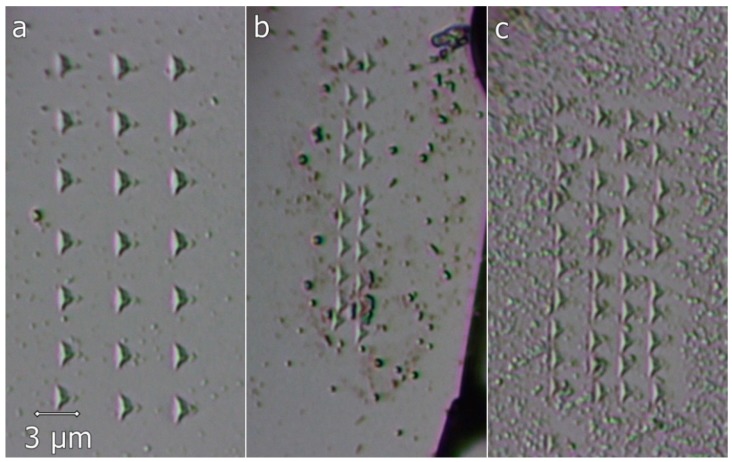
Instrumented indentation imprints on the thermally treated melt-spun ribbons: (**a**) Ti_60_Zr_10_Si_15_Nb_15_ ribbon—MS2; (**b**) Ti_64_Zr_10_Si_15_Nb_11_ ribbon—MS2; (**c**) Ti_56_Zr_10_Si_15_Nb_19_ ribbon—MS2.

Figure 12 represents the surface morphology of the thermally treated melt-spun ribbons, and the Berkovich indenter imprints performed in the material. One can observe that the material is homogeneous, due to the fact that the imprints exhibit almost identical shapes and sizes. Oxide traces are visible, mostly due to the polishing process. Crystalline grains cannot be observed for this magnification. Thus, the ribbons are retaining their structural refinement obtained due to the melt-spinning process, even after the heat treatment past the phase transformation temperature.

If we consider the measuring error related to the results from the bulk samples, it was shown that there is a direct correlation between the inhomogeneity of the material and the distribution of the values. Moreover, if we discuss the hardness of a material, several other factors can affect this characteristic. It might be assumed that the specimen material is originally stress-free prior to indentation. However, in many materials, stresses, tensile or compressive, may be present within the specimen as a result of processing, (temperature induced, as is the case of fast-cooling processes, thus related to the as-cast melt-spun ribbons, or thermal treatments, thus related to the heat treated ribbons) or sample surface preparation (cold working from mechanical polishing). The presence of residual stress can influence the results of instrumented indentation experiments, considering that the material recoils with different degrees (as a function of the type and level of internal stress), and therefore, it interacts differently with the diamond indenter.

In spite of the fact that the newly developed titanium alloys presented in the literature exhibit Young’s moduli relatively closer to that of the bone (the lowest value found in the literature was 14 GPa for a Ti–19 Nb–14 Zr (at. %) shape memory alloy [40]), and consist of highly compatible alloying elements, their wear resistance under loading conditions is reported to be still very poor.

The H/E ratio, (where H is the indentation hardness and E is the indentation elastic modulus), called the elastic strain to failure, gives information on the wear resistance of the material in question. Higher values for this ratio, meaning a combination of high hardness and low elastic modulus, would confer the material’s increased fracture toughness. Furthermore, the H^2^/E^2^ ratio gives information about the elastic resilience of materials (i.e., their ability to elastically absorb energy without yielding). Moreover, the H^3^/E^2^ ratio is an indicator regarding the material’s resistance against plastic deformation. Lower values of this ratio signify a poor resistance to plastic deformation.

Observing the results from Table 4, one can extract correlations which indicate certain predictions. It seems that the alloy variant with better wear resistance and better resistance to plastic deformation is the Ti_64_Zr_10_Si_15_Nb_11_ alloy, after melt-spinning in a vacuum and with a peripheral speed of 36 m/s, followed by thermal treatment past the phase transformation temperature and free cooling with the furnace. However, the metastable aspect of the proposed alloys would probably benefit primarily the manufacturing aspect, and to a lesser degree the final part properties, considering that the structure of the final material is affected on the one hand by the laser sintering followed by cooling (during additive manufacturing), and on the other hand by various treatments the part might be subjected to, for example, HIP (hot isostatic pressing).

### 3.3. Corrosion

#### 3.3.1. Potentiodynamic Studies

Due to the ionic composition of bodily fluids, a spontaneous process of corrosion can appear, which is able to destroy the surface of metallic implants. The chloride solutions are among the most aggressive and corrosive towards metals. Interactions between metals and the biological systems occur during electrochemical oxidation and reduction associated with corrosion processes. Electrochemical characterization of bulk alloy samples was performed through potentiodynamic studies and electrochemical impedance spectroscopy, to assess their corrosion resistance in environments that simulate biological fluids (NaCl 0.9% solution).

**Figure 13:**
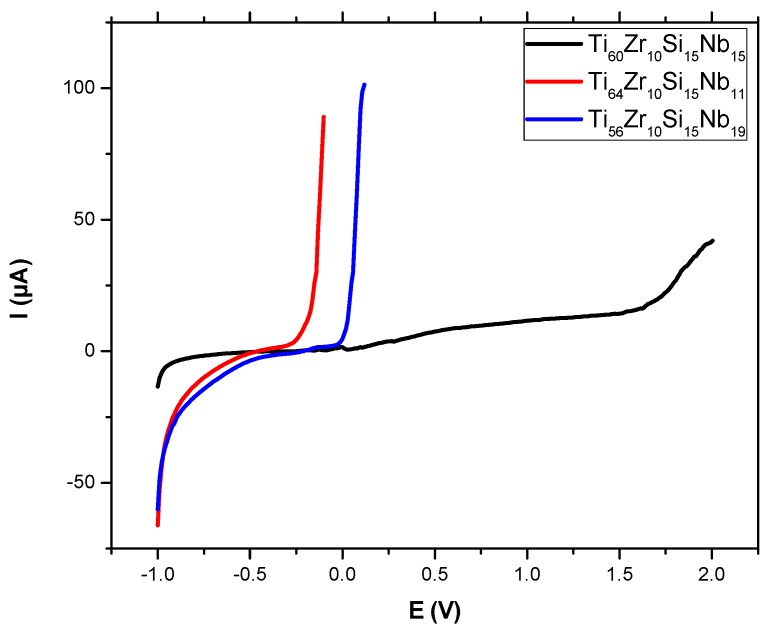
Polarization curves in NaCl 0.9% for bulk alloy samples.

**Figure 14:**
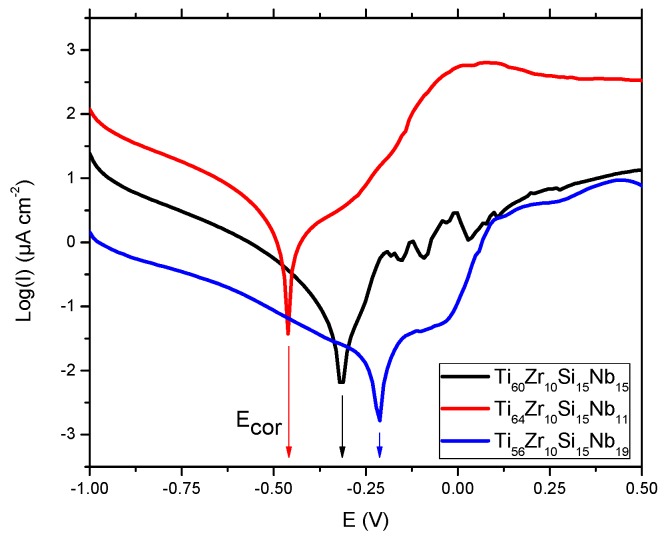
Evans diagrams in NaCl 0.9% for bulk alloy samples.

Potentiodynamic tests in NaCl 0.9% solution present an initial exponential growth trend of the current density when increasing the potential, as we can see on the representative plot in Figure 13 for bulk alloy sample Ti_60_Zr_10_Si_15_Nb_15_, with sensitive differences in corrosion parameters for the other two bulk alloy samples, presented in Table 5. These parameters are: the corrosion potential (E_corr_)—the potential at which the anodic and cathodic reaction rates are equal, the corrosion current density (J_corr_) as a measure of corrosion rate (v_corr_), polarization resistance (R_p_), passive domain E_pas_ (for which the anode current density keeps almost constant), the breakdown potential (E_bd_) obtained when a transpassive region was initiated and the anodic current density increased rapidly. The corrosion parameters were obtained from the corresponding polarization curves and Evans diagrams (Figure 14) and for this purpose, the Tafel slopes of cathodic and anodic branch were used and both Tafel slopes (βc and βa) were calculated.

The corrosion rate, v_corr_, in mm/year was calculated using PSTrace 5.5 software, where the equivalent weight was (EW) 99.99 g/mol, with density d = 7.00, 4.97, 5.3 g/cm^3^ and sample area (A) corresponding to each sample (0.0551, 0.0552, 0.0412 cm^2^) combined with a constant K = 3272 mm/(A cm year) (defined by the ASTM) (ASTM Standard G102-89, “Standard Practice for Calculation of Corrosion Rates and Related Information from Electrochemical Measurements,” Annual Book of ASTM Standards, ASTM International, West Conshohocken, Vol. 3.02, 2006).

In chloride containing solutions oxy-chloride compounds are resulting as pitting corrosion mechanisms. These ions act as activators of anodic reaction leading to a shift to more negative values of E_corr_. The sample is oxidizing and negative charges are accumulating in it. Usually, in metallic samples iron is the one which promotes the oxidation. As can be noticed in Table 5, for samples, Ti_60_Zr_10_Si_15_Nb_15_ and Ti_56_Zr_10_Si_15_Nb_19_ the corrosion potential E_corr_ was higher as compared with the standard redox potential of iron (−0.440 mV), while for sample Ti_64_Zr_10_Si_15_Nb_11_ is similar. However, an adherent, free of discontinuities oxide film (passivation film) may be formed in some situations on the samples’ surface.

The sample Ti_56_Zr_10_Si_15_Nb_19_ exhibited the smallest value of corrosion potential (−0.208 V) while the highest value was obtained on sample Ti_64_Zr_10_Si_15_Nb_11_. The corrosion current density had the biggest value for sample Ti_56_Zr_10_Si_15_Nb_19_ and the lowest for sample Ti_60_Zr_10_Si_15_Nb_15_ (0.26 μA/cm²). An identical value for the corrosion current density was reported elsewhere, for the Ti_75_Zr_10_Si_15_ alloy (0.26µA/cm^2^), while the addition of Nb, resulting in the Ti_60_Zr_10_Si_15_Nb_15_ alloy, was reported to lead to a 0.15µA/cm^2^ corrosion current density [15]. Decreasing of the corrosion current densities signifies that the corrosion rate of the sample is significantly reduced. Thus, for the corrosion rate, the general tendency of diminution has been noticed, with the smallest value of 0.012 mm/year for sample Ti_60_Zr_10_Si_15_Nb_15_. Similar values in cathodic Tafel slope, βc, have been observed for all bulk alloy samples, suggesting the same cathodic process mechanism. The influence of alloy type on the anodic Tafel slope, βa, indicates a modification in the mechanism of corrosion process (anodic reaction). The range of variation is related to sample surface homogeneity and composition of each alloy type. The highest value of βa was obtained for sample Ti_60_Zr_10_Si_15_Nb_15_, while the smallest one for sample Ti_56_Zr_10_Si_15_Nb_19_. Free corrosion behavior signifies no evidence of passivation layer forming on the sample surface. All passivation is inevitably followed by the breakdown of the passive film at the breakdown potential (E_bd_), beyond which corrosion increases steadily with increasing potential. An overall increasing of E_pass_ and E_bd_ starting with sample Ti_64_Zr_10_Si_15_Nb_11_, continuing with sample Ti_56_Zr_10_Si_15_Nb_19_ and sample Ti_60_Zr_10_Si_15_Nb_15_. Passivated metals often show metastable pitting corrosion before the real pitting corrosion starts. Current spikes in the polarization curve occur due to this phenomenon, as can be observed for sample Ti_60_Zr_10_Si_15_Nb_15_, which is the result of pit formation followed by pits passivation. Comparing all values, we can say that sample Ti_60_Zr_10_Si_15_Nb_15_ is more resistant to the corrosion process in NaCl 0.9% versus the remaining samples.

#### 3.3.2. Electrochemical Impedance Spectroscopy (EIS)

The experimental results obtained from EIS measurements for the corrosion of all bulk alloy samples in NaCl 0.9% solution are summarized in Table 6. This technique allows the possibility of studying corrosion reactions and measuring corrosion rates in low conductivity media, together with polarization resistance and double layer capacitance, simultaneously.

**Figure 15:**
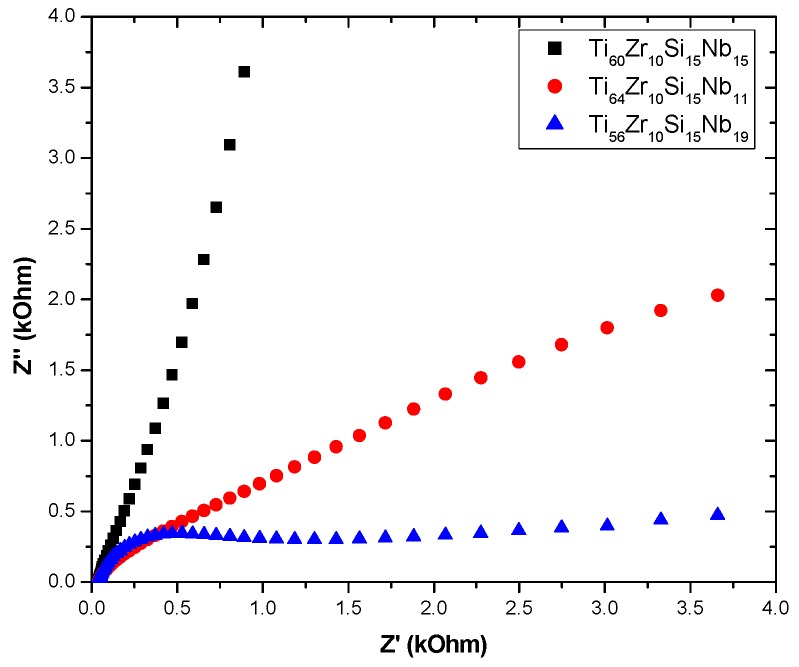
Nyquist impedance spectra in complex plane in NaCl 0.9% solution.

**Figure 16:**
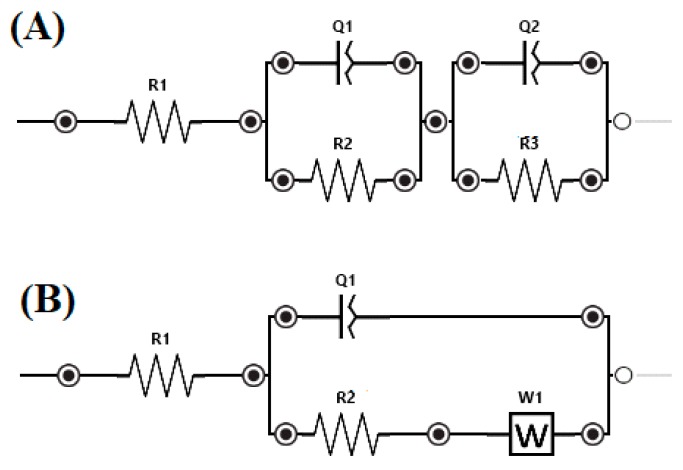
Equivalent electrical circuits for the bulk alloy samples: (**A**) For sample Ti_60_Zr_10_Si_15_Nb_15_ and Ti_64_Zr_10_Si_15_Nb_11_, (**B**) for sample Ti_56_Zr_10_Si_15_Nb_19_.

EIS measurements were performed using the AC signals of the 10 mV rms perturbation in the frequency range (f) 50 kHz–0.1 Hz, with 10 frequency values per frequency decade at the corrosion potential (specific to each sample from Table 5). Corresponding impedance spectra are presented as Nyquist plots in Figure 15. Figure 16 depicts the electrical equivalent circuits used to obtain the electrical parameters by fitting experimental EIS spectra, which can be used to describe the electrical features of the electrochemical interfaces between alloy samples and the electrolyte (NaCl 0.9 % solution). The equivalent circuits consist of the solution (cell) resistance, R_1_, in series with a one or two parallel R–CPE configuration which is attributed to the electrode (bulk alloy samples), and the passivation layer formed on its surface and charge-transfer process, respectively. R represents the charge transfer resistance of ions through electrochemical interfaces. The corroding surface of the electrode is expected to be inhomogeneous due to its roughness. Therefore, the capacitance is presented through a constant phase element (CPE, Z_CPE_ = 1/Q(iω)^n^). It was modeled as a non-ideal capacitor of capacitance Q (characterizing the double layer capacitance at the electrochemical interfaces and charge accumulation) and roughness factor, n, (n = 1 corresponds to a perfectly smooth surface), ω = 2πf. For the configuration with two parallel R–CPEs of the electrical equivalent circuit, the group [R_2_CPE_1_] has been used corresponding to an intermediate and low frequency, LF, while the parallel group [R_3_CPE_2_] was assigned to high-frequency region, HF. Moreover, we can say that R describes the charge transfer through passivated electrode-electrolyte interface, while Q and n characterize the double layer capacity and roughness/porosity of this interface.

**Figure 17:**
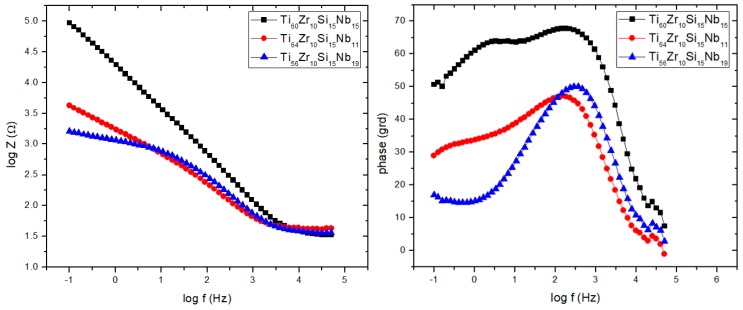
Bode plots for NaCl 0.9%.

By using the PSTrace 5.5 software with FRA module, the EIS experimental data were analyzed and the electric equivalent circuit best fitting the experimental data with electrical parameters was inferred. Different corrosion systems (e.g., charge transfer control, diffusion control or a mixture type) may show different features in the EIS spectra. Through analyzing the EIS data, and from the Nyquist plots (Figure 15) and Bode plots (Figure 17), the corrosion mechanism of the system can be identified.

Due to the possible formation of pores, the passive layer breakdown (pitting) would give rise to major differences in the impedance spectra. Thus, the decreasing of R values (resistance of the electrolyte filling such pores) due to the appearance of pores on the surface of the sample could occur. A circuit with one parallel R-CPE element in the series was necessary to fit the data in case of sample Ti_56_Zr_10_Si_15_Nb_19_, with a Warburg element added to describe the diffusion process of ions through the sample surface. For samples Ti_60_Zr_10_Si_15_Nb_15_ and Ti_64_Zr_10_Si_15_Nb_11_, a circuit with two parallel R-CPE elements in series was used, indicating a charge transfer process controlling the corrosion of the sample, due to rapid appearance of the pitting pores in the passivation layer on top of the sample Ti_64_Zr_10_Si_15_Nb_11_ and charge accumulation at the interfaces, especially for sample Ti_64_Zr_10_Si_15_Nb_11_ (data in accordance with the results from potentiodynamic studies). The degradation mechanism can be related to the water saturation of the free volume of the sample surface, followed by the electrolyte penetration into the metal-layer interface that intensified the corrosion process (decreasing values of R_2_).

Direct information about impedance, frequency and phase, can be obtained from Bode plots (Figure 17) that helps to determine and correlate the different constituent phases of the system with the data from the Nyquist plots. In all experimental situations the Bode plots show that in the high-frequency region, a capacitive behavior is predominant for samples Ti_64_Zr_10_Si_15_Nb_11_ and Ti_56_Zr_10_Si_15_Nb_19_, with resistive behavior for sample Ti_60_Zr_10_Si_15_Nb_15_. The same capacitive behavior has been noticed for the low-frequency region range, for samples Ti_60_Zr_10_Si_15_Nb_15_ and Ti_64_Zr_10_Si_15_Nb_11_, while for sample Ti_56_Zr_10_Si_15_Nb_19_ the diffusional behavior was predominant. Two defined time constants characterize the data for samples Ti_60_Zr_10_Si_15_Nb_15_ and Ti_64_Zr_10_Si_15_Nb_11_ from Table 5. This behavior suggested that two processes take place simultaneously, at the alloy—passive film interface and at the passive film—NaCl solution interface. Thus, in the phase angle vs. log frequency curve, in the low and the intermediate frequency range, two well-defined maxima that correspond to the phase angles of approximately 60 and 65 grd. appear, which indicates capacitive behavior of sample Ti_60_Zr_10_Si_15_Nb_15_. This suggests the existence of a highly passive stable film on the surface, with characteristics close to pure capacitive impedance. For the sample Ti_64_Zr_10_Si_15_Nb_11_ a similar behavior was noticed, but the two maxima are not so well defined and correspond to smaller phase angles of approximately 30 and 45 grd., which means that on the sample surface a very stable passive film is not formed, to hinder the charge transfer reaction. These observations are in accordance with the potentiodynamic polarization behavior. The situation is similar for sample Ti_56_Zr_10_Si_15_Nb_19_.

Comparing all data from EIS measurements, it can be concluded that sample Ti_60_Zr_10_Si_15_Nb_15_ is more resistant to the corrosion process in NaCl 0.9% solution, as compared with the other two bulk alloy samples.

### 3.4. Thermal Analysis

**Figure 18:**
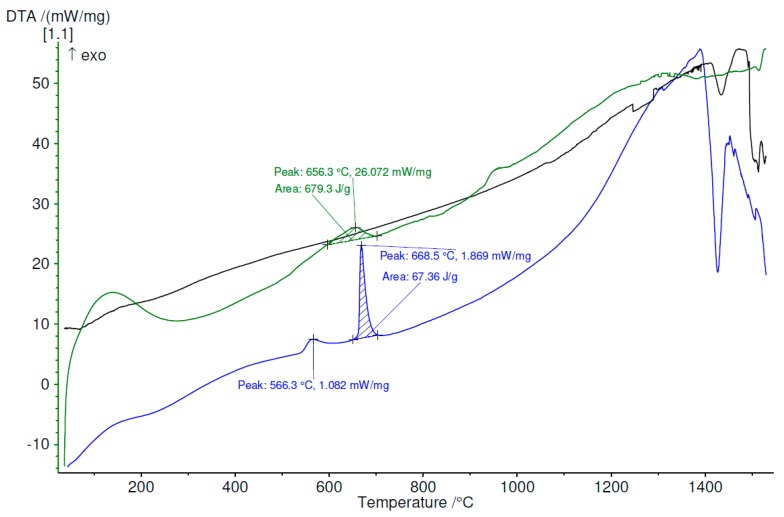
DTA variation as a function of temperature, up to 1530 °C, for Ti_56_Zr_10_Si_15_Nb_19_ alloy, bulk (black) and melt-spun ribbons (green—MS1, blue—MS2).

**Figure 19:**
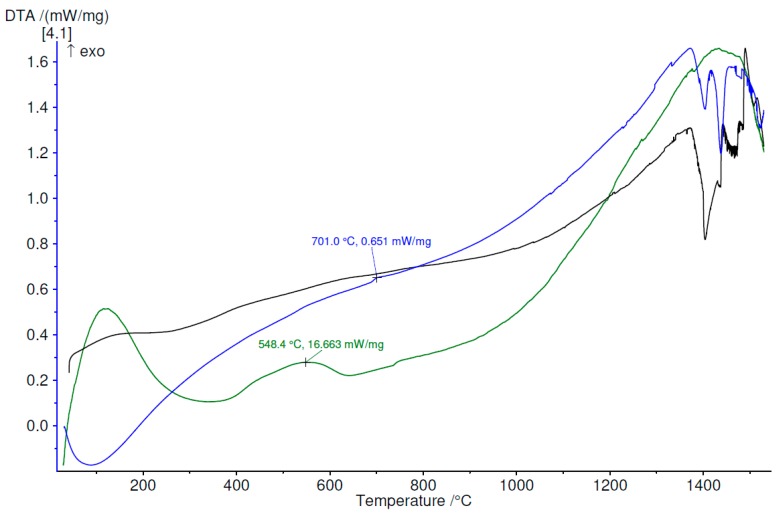
DTA variation as a function of temperature, up to 1530 °C, for Ti_60_Zr_10_Si_15_Nb_15_ alloy, bulk (black) and melt-spun ribbons (green—MS1, blue—MS2).

**Figure 20:**
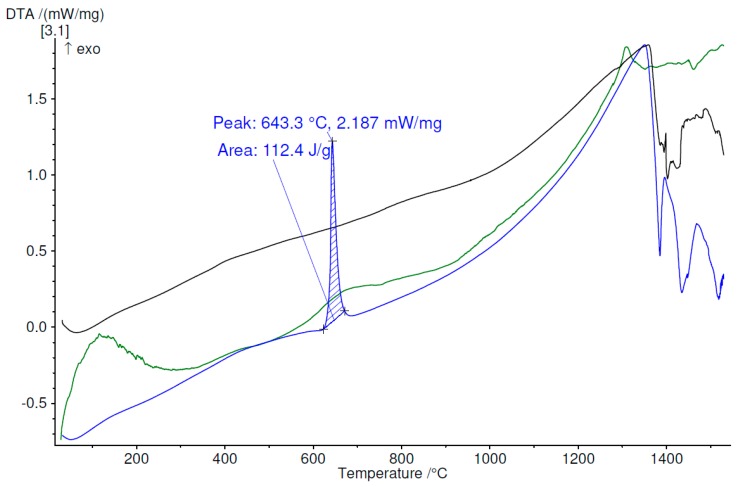
DTA variation as a function of temperature, up to 1530 °C, for Ti_64_Zr_10_Si_15_Nb_11_ alloy, bulk (black) and melt-spun ribbons (green—MS1, blue—MS2).

The thermal stability and crystallization temperatures were measured by differential thermal analysis. The analyses, performed in argon atmosphere, up to 1530 °C for each set of melt-spun ribbons and also for the bulk material, demonstrate that exothermal processes appear in all cases of melt-spun ribbons, in contrast with the bulk alloys, where no exothermal reaction was observed. With the increase in the percentage of the amorphous phase, the tendency to produce exothermic processes grows. Moreover, even if some of the melt-spun ribbons are not entirely amorphous, but nanostructured, they exhibit exothermal reactions, to a lesser degree, all in the 550–800 °C region. Above 1300 °C, all samples, regardless of processing characteristics, start to partially melt, as can be observed in Figure 18, Figure 19 and Figure 20, for the Ti_56_Zr_10_Si_15_Nb_19_ alloy, the Ti_60_Zr_10_Si_15_Nb_15_ alloy, and the Ti_64_Zr_10_Si_15_Nb_11_ alloy variants, respectively.

The difference in behavior during heating, as a function of the cooling rate, which was obtained during the melt spinning processes, can be observed in Figure 18 for the Ti_56_Zr_10_Si_15_Nb_19_ alloy. The MS1 melt-spun ribbon exhibits one broad peak with the maximum at 656 °C. The presence of this broad peak certifies that a certain degree of amorphization or structural refinement occurs during melt spinning, even if the cooling rate is not as high as the one obtained during the MS2 castings. In the case of the MS2 melt-spun ribbon, two exothermal peaks, located at 566 °C and 668.5 °C can be observed, the latter with a significantly higher intensity. The curve representing the variation of DTA as a function of temperature for the bulk Ti_56_Zr_10_Si_15_Nb_19_ alloy does not exhibit any exothermal peaks. This observation should be an indication regarding the stable (bulk) or metastable (melt-spun ribbons) characteristic of the alloy, as a function of the processing parameters. Over 1300 °C, the eutectic is no longer stable, thus the melting process of this phase can be inferred from the endothermal peaks, visible on the DTA curves. Similar behavior is exhibited by the samples from the Ti_64_Zr_10_Si_15_Nb_11_ alloy: the MS1 melt-spun ribbon exhibits a broad band starting with 600 °C, the MS2 ribbon exhibits a sharp exothermal peak with the maximum at 643.3 °C, the bulk alloy has a stable structure, starting to melt after 1300 °C. Regarding the Ti_60_Zr_10_Si_15_Nb_15_ alloy, the variation of the DTA as a function of the temperature is significantly different, compared to the remaining compositions. In the case of the MS1 ribbon, instead of a sharp exothermal peak, which would have been expected after 600 °C, a broad band is visible. This observation leads to the conclusion that the cooling rate during melt-spinning is not sufficient for complete amorphization, however, a certain degree of the amorphous phase is still present. This behavior is in agreement with the structural analysis.

## 4. Conclusions

New biocompatible Ti-based amorphous and nanocrystalline metallic structures with various compositions (variable Ti and Nb percentages, constant Zr and Si percentages) were obtained by a melt-spinning process with different cooling rates. The structure of the melt-spun alloys depends on the alloy composition and on the cooling rate provided by the melt-spinning process. Although amorphous ribbons were obtained only for two compositions (Ti_56_Zr_10_Si_15_Nb_19_ and Ti_64_Zr_10_Si_15_Nb_11_) a structural refinement is noticed for all the other compositions, with a crystallite size for the predominant β phase smaller than 21 nm. Moreover, the amorphous Ti_64_Zr_10_Si_15_Nb_11_ variant could exhibit better resistance to wear and to plastic deformation, inferred from the instrumented indentation results. The potentiodynamic polarization curves performed in saline solution showed that for the alloy sample Ti_60_Zr_10_Si_15_Nb_15_ a stable passive layer was formed. This alloy has passive behavior on a large potential range. For the remaining alloy compositions, relatively instable passive films have been formed, in very small potential ranges. At more positive potentials, for all alloy samples, the current densities increase again, due to both transpasivation and oxygen evolution reaction. Thermal analysis shows that exothermic processes appear for all melt-spun ribbons (unlike the bulk alloys) but at a higher intensity with the increase of the amorphous phase percentage. As a result, lower input power during additive manufacturing processing of the studied biocompatible Ti-based alloys could be needed.

## Figures and Tables

**Table 1:** Composition of Ti-based alloys.

Alloy		Ti	Zr	Si	Nb
Ti_60_Zr_10_Si_15_Nb_15_	[% at]	60	10	15	15
	[% wt]	51.29	16.29	7.52	24.89
Ti_64_Zr_10_Si_15_Nb_11_	[% at]	64	10	15	11
	[% wt]	56.53	16.83	7.77	18.86
Ti_56_Zr_10_Si_15_Nb_19_	[% at]	56	10	15	19
	[% wt]	46.38	15.78	7.29	30.54

**Table 2:** Crystallite size for the melt-spun ribbons, estimated with the Scherrer equation.

Process	Alloy	Crystallite Size (nm)
MS1	Ti_60_Zr_10_Si_15_Nb_15_	17
	Ti_64_Zr_10_Si_15_Nb_11_	18
	Ti_56_Zr_10_Si_15_Nb_19_	21
MS2	Ti_60_Zr_10_Si_15_Nb_15_	21
	Ti_64_Zr_10_Si_15_Nb_11_	0 (amorphous)
	Ti_56_Zr_10_Si_15_Nb_19_	0 (amorphous)

**Table 3:** Chemical composition as function of phase.

Alloy	Acquisition Site	Chemical Composition [% at]			
		Ti	Si	Zr	Nb
bulk Ti_60_Zr_10_Si_15_Nb_15_	spectrum 1	85.61	0.42	5.39	8.57
	spectrum 2	61.56	16.21	16.64	5.59
bulk Ti_64_Zr_10_Si_15_Nb_11_	spectrum 1	40.60	37.64	14.55	7.20
	spectrum 2	78.39	1.76	4.58	15.28
bulk Ti_56_Zr_10_Si_15_Nb_19_	spectrum 1	57.84	39.70	2.29	0.181
	spectrum 2	86.71	11.28	1.74	0.27
Ti_60_Zr_10_Si_15_Nb_15_ ribbons MS1	spectrum 1	76.53	2.69	8.14	12.65
	spectrum 2	65.30	12.73	12.31	9.66
Ti_64_Zr_10_Si_15_Nb_11_ ribbons MS1	spectrum 1	59.42	17.60	10.51	12.46
	spectrum 2	44.83	31.68	13.00	10.49
Ti_56_Zr_10_Si_15_Nb_19_ ribbons MS1	spectrum 1	59.99	13.38	10.03	16.61
	spectrum 2	42.48	31.45	14.23	11.84

**Table 4:** Mechanical properties of the Ti-based alloys, either in bulk form or in melt-spun ribbon form.

Alloy	Variant	H (GPa)	E (GPa)	H/E	H^2^/E^2^	H^3^/E^2^
Ti_56_Zr_10_Si_15_Nb_19_	bulk	7.23 ± 2.89	120.04 ± 20.21	0.0602	0.0036	0.0262
	MS1	5.41 ± 0.51	87.35 ± 3.44	0.0620	0.0038	0.0208
	MS2	7.71 ± 0.99	102.08 ± 5.05	0.0755	0.0057	0.0440
	MS2 + TT	5.73 ± 1.06	97.23 ± 10.11	0.0589	0.0035	0.0199
Ti_60_Zr_10_Si_15_Nb_15_	bulk	6.06 ± 0.56	125.52 ± 7.46	0.0484	0.0023	0.0142
	MS1	7.00 ± 1.51	94.16 ± 9.55	0.0744	0.0055	0.0388
	MS2	6.66 ± 0.35	103.68 ± 2.99	0.0643	0.0041	0.0276
	MS2 + TT	8.31 ± 0.78	125.16 ± 5.30	0.0665	0.0044	0.0368
Ti_64_Zr_10_Si_15_Nb_11_	bulk	4.73 ± 0.92	122.54 ± 15.97	0.0387	0.0015	0.0071
	MS1	7.63 ± 1.52	127.19 ± 12.19	0.0600	0.0036	0.0275
	MS2	6.21 ± 0.54	118.50 ± 7.90	0.0524	0.0027	0.0171
	MS2 + TT	9.92 ± 0.28	127.89 ± 3.02	0.0776	0.0060	0.0597

**Table 5:** Corrosion parameters in NaCl 0.9%.

Sample/Parameter	E_corr_ [V]	I _corr_ [μA]	J_corr_ [μA/cm²]	R_p_ [kΩ]	β_a_ [V/decade]	β_c_ [V/decade]	v_corr_ [mm/year]	E_pass_ [V]	E_bd_ [V]
Ti_60_Zr_10_Si_15_Nb_15_	−0.327	0.143	0.260	616.70	0.411	0.399	0.012	2.10	1.60
Ti_64_Zr_10_Si_15_Nb_11_	−0.437	0.662	1.199	78.59	0.256	0.370	0.079	0.25	−0.25
Ti_56_Zr_10_Si_15_Nb_19_	−0.208	0.537	1.302	90.57	0.160	0.373	0.804	0.40	0.00

**Table 6:** Electrochemical impedance spectroscopy (EIS) results.

Sample/Parameter	R 1	Q 1	n 1	R 2	Q 2	n 2	R 3	W1
	Ω cm^2^	μF^−1^ cm^−2^ s^−n^		Ω cm^2^	μF^−1^ cm^−2^ s^−n^		Ω cm^2^	KΩ s^−0.5^
Ti_60_Zr_10_Si_15_Nb_15_	1.18	874.77	0.74	45.07	466.24	0.76	4536.16	-
Ti_64_Zr_10_Si_15_Nb_11_	1.50	4327.90	0.59	438.05	1531.27	0.70	31.14	-
Ti_56_Zr_10_Si_15_Nb_19_	1.39	-	-	-	638.04	0.75	40.98	441.00
